# Long-Term, Competitive Swimming and the Association with Atrial Fibrillation

**DOI:** 10.1186/s40798-016-0066-8

**Published:** 2016-10-17

**Authors:** Andrew D. Schreiner, Brad A. Keith, Karen E. Abernathy, Jingwen Zhang, Walter A. Brzezinski

**Affiliations:** Division of General Internal Medicine, Department of Medicine, Medical University of South Carolina, 135 Rutledge Avenue, Suite 1240, Charleston, SC 29425 USA

**Keywords:** Endurance exercise, Swimming, Competitive swimming, Atrial fibrillation

## Abstract

**Background:**

Endurance exercise plays a role in cardiovascular risk reduction, but may also be a risk factor for atrial fibrillation. This study was performed to assess the prevalence of atrial fibrillation in a population of long-term, competitive swimmers compared with patients within an internal medicine clinic with known risk factors for atrial fibrillation such as diabetes mellitus and hypertension.

**Methods:**

This cross-sectional study utilized survey data comparing the prevalence of atrial fibrillation in swimmers to a general internal medicine population. A multi-national group of swimmers over the age of 60 were surveyed, and a chart review was performed on a random sample of age-matched internal medicine patients. The primary outcome was the diagnosis of atrial fibrillation. Univariate analysis was used for means of proportions of the responses, and a multivariate logistic regression analysis was performed with diagnosis of atrial fibrillation as the dependent variable.

**Results:**

Forty-nine swimmers completed surveys and 100 age-matched internal medicine patients underwent chart review. Swimmers reported atrial fibrillation in 13 cases (26.5 %) compared to 7 (7 %) in the comparison group (*p* = 0.001). A diagnosis of hypertension or diabetes mellitus was present in 23 (46.9 %) and 1 (2 %) of the swimmers, respectively, as compared to 72 (72 %, *p* = 0.003) and 32 (32 %, *p* < 0.001) in the comparison group. Age, presence of diabetes mellitus, and swimming history were variables included in the logistic regression, in relation to atrial fibrillation. Swimming was associated with an odds ratio of 8.739 (95 % CI 2.290 to 33.344, *p* = 0.015).

**Conclusions:**

Long-term, competitive swimmers have an increased prevalence of atrial fibrillation compared to internal medicine patients, despite the higher burden of diabetes mellitus and hypertension in the internal medicine group.

**Electronic supplementary material:**

The online version of this article (doi:10.1186/s40798-016-0066-8) contains supplementary material, which is available to authorized users.

## Key Points

- An association exists between high-intensity exercise over time and the diagnosis of atrial fibrillation.
- Long-term, competitive swimmers had a prevalence of atrial fibrillation exceeding a comparison group with a significantly higher burden of known cardiovascular risk factors, including diabetes mellitus and hypertension.
- Identifying a threshold of risk for atrial fibrillation in endurance athletes may provide clinicians with better information to counsel patients on the risks and benefits of exercise.

## Background

Atrial fibrillation is one of the most common cardiac arrhythmias. Its prevalence increases with age and affects nearly 5 % of patients over the age of 65. Known risk factors include age, hypertension, diabetes mellitus, and structural heart disease [1]. While endurance exercise plays an integral role in reducing cardiovascular risk, multiple studies have identified an association between prolonged periods of high-intensity exercise and the diagnosis of atrial fibrillation [2–7]. Prior research has shown that participants in athletic endeavors including marathon running, cycling, and cross-country skiing have an increased prevalence of atrial fibrillation [8–10]. The purpose of this study is to assess the prevalence of atrial fibrillation in a population of long-term, competitive swimmers. Further, it aims to compare this prevalence to a group of patients from an academic internal medicine clinic, and many of whom have known atrial fibrillation risk factors including diabetes mellitus and hypertension.

## Methods

This cross-sectional study assesses the prevalence of atrial fibrillation in two groups. The first group, the US Masters Swim Forum (“The Swim Forum”), comprises an international assemblage of male and female long-term, competitive swimmers over the age of 60. The group includes former Olympians and swimmers holding world records in their respective age groups. Membership is by invitation only with subsequent registration on an internet-based forum. At the time of this study, the “Swim Forum” had 96 registered members.

The comparison group consists of patients within an academic internal medicine clinic at the Medical University of South Carolina (MUSC). Age-matched patients were selected from the clinic population, which includes over 12,000 medically and racially diverse patients. The demographic characteristics of the clinic’s patient population include a mean age of 57, with 17 % of patients 70 years of age or older, 37 % males and nearly half are African-American (47 %).

In 2015, all Swim Forum registrants received an electronic invitation to participate in a web-based survey. Informed consent accompanied the survey, and three electronic announcements encouraged members to respond. Intentionally brief to maximize participation, the survey queried Swim Forum members if they had ever been diagnosed with atrial fibrillation, diabetes mellitus, or hypertension (Additional file 1). The survey was originally intended to determine the prevalence of atrial fibrillation in the Swim Forum. Questions included “Have you ever been diagnosed with atrial fibrillation?”, “Do you have diabetes?” and “Have you ever been diagnosed with or are currently being treated for high blood pressure (hypertension)?” Survey participants self-reported these diagnoses. Given the confidentiality of the survey, we were unable to identify those Swim Forum members who did not complete it, and thus could not provide further encouragement for them to do so.

Of the 96 Swim Forum members, 49 completed the survey and an age-matched comparison group was randomly selected from the internal medicine clinic at a ratio of 2:1, for a total of 100 patients. Data collection occurred through chart review, utilizing the survey tool provided to the swimmers. Data were obtained from the academic internal medicine clinic’s electronic medical record (EPIC) and relied upon the patients’ problem lists, past medical histories, and past medication histories. Aside from age-matching, there were no exclusion criteria, and members of the internal medicine group were assumed to not swim competitively. This study received approval from the Internal Review Board at the Medical University of South Carolina and was conducted in accordance with the Helsinki Declaration.

The primary outcome measure was the diagnosis of atrial fibrillation via self-report in the swimmers and chart review in the internal medicine patients. Univariate analysis (two sample *t* test and Chi-square test) was used for means of proportions of the survey responses. Additionally, a multivariable logistic regression analysis was performed with diagnosis of atrial fibrillation as the dependent variable. Covariates were tested for a significance level of *p* < 0.10 via a backward selection process. The goodness of fit was assessed by the Hosmer and Lemeshow test and receiver operating characteristic (ROC) curve, which was created by plotting sensitivity against (1- specificity) to establish the accuracy of predictions. The area under the ROC curve (AUC) was used to determine the quality of predictors. SAS 9.3 (SAS Institute Inc., Cary, NC) was used for statistical analyses.

## Results

Forty-nine swimmers responded to the survey (response rate of 51 %), with an average age of 72.6 years (SD of 4.9 years). In the comparison group, 100 age-matched patients were randomly selected, resulting in an average age of 70.9 years (*p* = 0.074). Table 1 reports the patient characteristics of the swimmers and non-swimmers based on compiled survey responses and chart review, respectively. Swim Forum members reported a diagnosis of atrial fibrillation in 13 cases (26.5 %) compared to 7 cases (7 %) in the comparison group (*p* = 0.001). A diagnosis of hypertension or diabetes mellitus was present in 23 (46.9 %) and 1 (2 %) of the swimmers, respectively, as compared to 72 (72 %, *p* = 0.003) and 32 (32 %, *p* < 0.001) in the comparison group. There was no significant difference in the use of statin, aspirin, or anticoagulation medication between the two groups. The number of patients with cerebrovascular disease diagnoses (either cerebrovascular accident (CVA) or transient ischemic attack (TIA)) was similar between swimmers (10.2 %) and non-swimmers (10 %).

**Table 1:** Patient characteristics

	Swimmers (*N* = 49)	Non-swimmers (*N* = 100)	*p* value
Age, year (mean ± SD)	72.6 ± 4.9	70.9 ± 6.4	0.074
Years swimming (mean ± SD)	25.6 ± 16.9	0 ± 0	< 0.001
Weekly swimming mileage (mean ± SD)	6.0 ± 3.8	0 ± 0	< 0.001
Atrial fibrillation, no. (%)	13 (26.5)	7 (7.0)	0.001
Hypertension, no. (%)	23 (46.9)	72 (72.0)	0.003
Diabetes mellitus, no. (%)	1 (2.0)	32 (32.0)	< 0.001
Cerebrovascular disease, no. (%)	5 (10.2)	10 (10.0)	1.000
Anticoagulation, no. (%)	6 (12.2)	8 (8.0)	0.390
Aspirin, no. (%)	29 (59.2)	48 (48.0)	0.199
Statin, no. (%)	26 (53.1)	66 (66.0)	0.127
Previous heart surgery, no. (%)	12 (24.5)	7 (7.0)	0.003
Venous thromboembolism, no. (%)	0 (0.0)	2 (2.0)	1.000

**Fig. 1:**
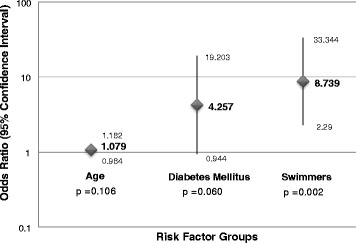
Age, diabetes mellitus, and swimming were included in the final model (*c* = 0.768). Swimming was the only covariate to have a statistically significant association with atrial fibrillation (*p* = 0.002), with an odds ratio of 8.739 (95 % CI 2.290 to 33.344). Diabetes mellitus trended toward significance with a *p* value of 0.06

Using logistic regression analysis, potentially predictive covariates (age, history of competitive swimming, and the presence of hypertension or diabetes mellitus) were analyzed, with the diagnosis of atrial fibrillation as the dependent variable. Age, diabetes mellitus, and swimming were included in the final model (*c* = 0.768). Swimming was the only covariate to have a statistically significant association with atrial fibrillation (*p* = 0.002), with an odds ratio of 8.739 (95 % CI 2.290 to 33.344). Diabetes mellitus trended toward significance with a *p* value of 0.06. Odds ratios are further illustrated in Fig. 1.

## Discussion

This study highlights the high prevalence of atrial fibrillation among long-term, competitive swimmers. Our findings are supported by previous studies that have found an increased prevalence of atrial fibrillation in endurance athletes [3, 7]. This suggests that competitive swimming should be included in the list of aerobic activities associated with atrial fibrillation, along with long-distance running, cross-country skiing, and cycling. The prevalence of atrial fibrillation reported by the Swim Forum was 26.5 %, far exceeding that of the 7 % in the comparison group. This disparity is particularly compelling as the internal medicine patients had a much higher burden of hypertension and diabetes mellitus, which are known risk factors for atrial fibrillation. These data from the comparison group are in accordance with the reported prevalence of atrial fibrillation (5 %) in the general population age 65 or older previously described in the literature [1].

The swimmers’ self-reported prevalence of atrial fibrillation surpasses what has been identified in other research regarding the link between endurance exercise and atrial arrhythmia. One example of this difference is the 12.8 % prevalence of atrial fibrillation identified by Grimsmo and colleagues in cross-country skiers, as confirmed by electrocardiogram [9]. A potential explanation for this disparity is selection bias, as swimmers with atrial fibrillation may have been more likely to respond to the survey. In order to protect confidentiality, we were unable to further investigate non-respondents. However, even if one assumes that all non-respondents do not possess the diagnosis of atrial fibrillation, the prevalence in the swimmer group would remain elevated at 13.5 % (similar to the prevalence identified by Grimsmo). Another consideration for the high prevalence of atrial fibrillation in the Swim Forum may relate to the members’ elite level of performance and training. Although skiers in the aforementioned study were top quartile performers by age, the Swim Forum consists of swimmers at the leading edge of competition with ex-Olympians, world record holders, and national record holders by age group. If an appreciable difference exists between the intensity and duration of training for the skiers and swimmers, it may suggest a threshold of physical activity beyond which the association with atrial fibrillation escalates.

Although regular exercise is well studied and has been shown to decrease cardiovascular risk, there is an emerging body of literature supporting the concept for a threshold in the association of atrial fibrillation and high-intensity exercise. Elosua and colleagues studied cases of lone atrial fibrillation and found substantially more of the patients with atrial fibrillation participated in regular sport activity than was observed in the general population. Further, they posited the threshold for increased risk to be 1500 lifetime hours of practice [11]. Myrstad and colleagues also found evidence to support a threshold for risk in studying cross-country skiers compared to a general population [12]. Another recent study reported the presence of a “U-shaped curve” in the association between high-intensity exercise and atrial fibrillation. The prevalence of atrial fibrillation increased for study participants with greater than 2000 lifetime hours of intense physical activity, whereas those with fewer than 2000 lifetime hours of activity had a lower prevalence of atrial fibrillation in comparison with sedentary individuals [13]. While members of the Swim Forum reported current swimming patterns, the survey did not solicit lifelong accumulated hours of high-intensity exercise. Identifying the threshold of endurance exercise beyond which patients are more at risk for developing atrial fibrillation is an important challenge, and further research is needed to provide recommendations for patients, particularly athletes.

The association of atrial arrhythmias with endurance exercise and the underlying pathophysiology remains a topic of debate. Studies contend that fibrotic change, atrial remodeling, and increased vagal tone are involved in the relationship between arrhythmia and exercise [14–16]. While investigating these mechanisms was beyond the scope of this study, future research may play a pivotal role in assessing the risk of arrhythmia in endurance athletes.

Several limitations of this study should be considered. The swimmers’ self-report of atrial fibrillation by survey introduces the potential for selection bias. Fifty-one percent of the Swim Forum registered members responded to the survey, which could falsely elevate the prevalence of atrial fibrillation in the population. Although 96 swimmers are registered, it is not known how many members maintain an active role in the forum. Non-respondents could not be characterized due to the anonymity of collected data. The use of self-report may also lead to misclassification of exposures and outcomes by respondents, potentially resulting in under-reporting of hypertension and diabetes mellitus in the swimmers. In comparison, data collection for the internal medicine patients relied upon chart review. Inherent differences in the swimmers and internal medicine patients and the brevity of the survey tool may contribute to confounding, as it is likely the groups differ in predictor variables, both known and unknown. Specifically, gender was not evaluated. A control group consisting of healthy individuals of similar age who did not engage in swimming may better isolate the association of swimming with atrial fibrillation. Lastly, it may be overzealous to assume the internal medicine group does not engage in endurance swimming. However, due to the intensity of exercise and the achievement necessary for inclusion in the Swim Forum, disparities in the magnitude of swimming likely exist.

## Conclusions

Regular exercise provides many benefits to cardiovascular health; however, long-term high-intensity exercise may pose an increased risk of atrial fibrillation. This study highlights the significant difference in prevalence of atrial fibrillation between elite swimmers and a group of internal medicine patients. Even though the internal medicine group had more risk factors due to a higher rate of diabetes mellitus and hypertension, the prevalence of atrial fibrillation in swimmers was remarkably higher than of internal medicine patients. These findings warrant further investigation into the cardiovascular risks of endurance athletes.

## Additional file

Additional file 1: Atrial fibrillation in endurance athletes study. (PDF 39 kb)
