# Supplementary material for: Long-Term, Competitive Swimming and the Association with Atrial Fibrillation
Source: Sports Med Open. 2016 Oct 17;2:42. doi: 10.1186/s40798-016-0066-8 (PMC5067262; doi:10.1186/s40798-016-0066-8)
Supplement: Additional file 1: — Atrial fibrillation in endurance athletes study. (PDF 39 kb) [file 40798_2016_66_MOESM1_ESM.pdf]

Atrial Fibrillation in Endurance Athletes Study

We are conducting a survey to evaluate the incidence of atrial fibrillation in endurance swimmers. The purpose of this research is to further identify the presence of a particular cardiac rhythm disturbance, atrial fibrillation, in long distance swimmers. Future studies may look to see if long term swimming has any impact on this condition. If you choose to participate, you will be asked to complete this twelve question survey, which should take approximately five minutes. There are no further questions, testing, or interventions included. Participation in this survey is voluntary, and participants are entitled to withdraw at any time. The information gathered will not contain identifiable information and will not be linked to the survey participant. This study is purely observational, and will not involve any form of intervention or treatment. If you have any questions regarding the study, the survey, or any other concerns, please contact the research coordinator, Dr. Andrew Schreiner, at 843-876-8688, or at schrein@musc.edu. Your response is greatly appreciated.

1) What is your age? ________________________________

2) Approximately how many years have you been involved in distance swimming? ________________________________

3) How many miles per week do you currently swim? ________________________________

4) Have you ever been diagnosed with atrial fibrillation?
   ◯ Yes
   ◯ No

5) Have you ever been diagnosed, or are currently being treated for high blood pressure (Hypertension)?
   ◯ Yes
   ◯ No

6) Do you have diabetes?
   ◯ Yes
   ◯ No

7) Have you ever been diagnosed with a stroke or transient ischemic attack (TIA)?
   ◯ Yes
   ◯ No

8) Do you currently take a blood thinner or anticoagulant medication (i.e. warfarin, enoxaparin, rivaroxaban, or apixiban)?
   ◯ Yes
   ◯ No

9) Do you currently take aspirin?
   ◯ Yes
   ◯ No

10) Are you currently on a cholesterol medication (statin)?
   ◯ Yes
   ◯ No

11) Have you ever had surgery on your heart?
   ◯ Yes
   ◯ No

12) Have you ever had a blood clot to your lungs?
   ◯ Yes
   ◯ No
